# Supplementary figures and images for: Is price associated with the quality of medicines? Evidence from active pharmaceutical ingredient testing in Nigeria
Source: PLoS One. 2025 Dec 15;20(12):e0338739. doi: 10.1371/journal.pone.0338739 (PMC12704850; doi:10.1371/journal.pone.0338739)

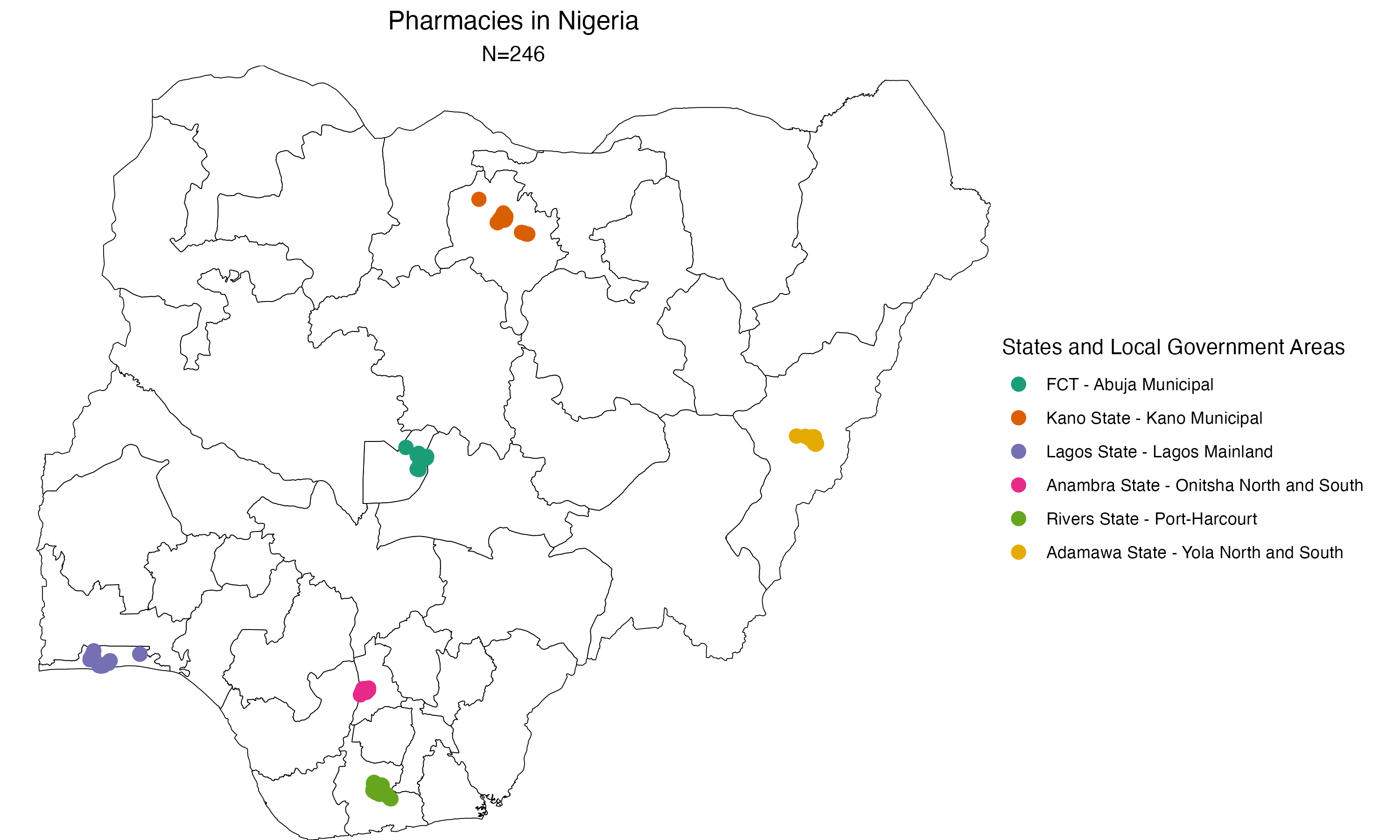

Supplement: S1 Fig — Notes: National Overview. Geographic distribution of sampled pharmacies across Nigeria, stratified by study state. Each color represents one of the six sampled states. Dots correspond to individual pharmacy locations included in the study. Maps created by the authors using public data from Natural Earth. (PNG) [file pone.0338739.s001.png]

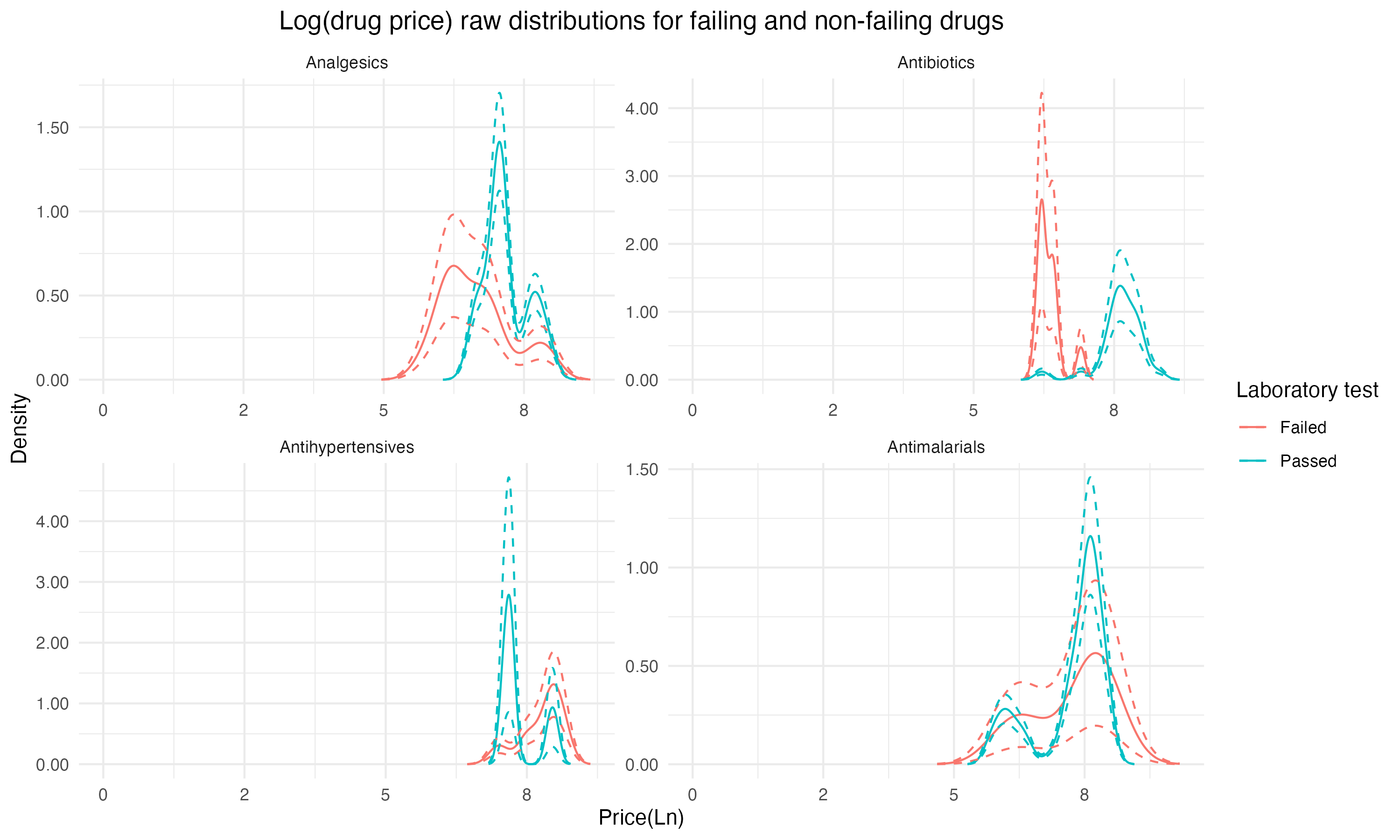

Supplement: S3 Fig — Notes: The figure shows the kernel densities of price (ln, in Nigerian Naira and in US dollars) for drug samples (n = 246) that passed or failed the laboratory test, by category of medicines: analgesics, antibiotics, antihypertensives and antimalarials. Dotted lines represent the 95% confidence intervals. (PNG) [file pone.0338739.s003.png]

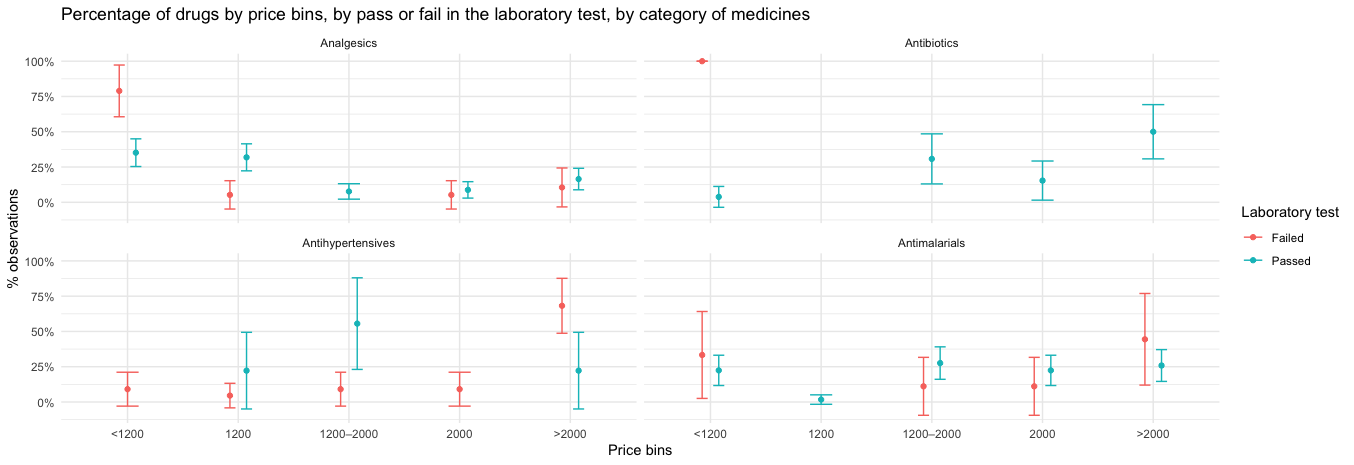

Supplement: S4 Fig — Notes: This figure shows the distribution of laboratory test outcomes across five mutually exclusive price categories: Nigerian Naira NGN 2000 or USD 4.6, by category of medicines: analgesics, antibiotics, antihypertensives and antimalarials. Bins lines represent the 95% confidence intervals. Exchange rate at November 30, 2022 was Nigerian Naira 438.6 = USD 1 from Nigerian Central Bank: https://www.cbn.gov.ng/rates/ExchRateByCurrency.html. (PNG) [file pone.0338739.s004.png]
