# Supplementary material for: Is price associated with the quality of medicines? Evidence from active pharmaceutical ingredient testing in Nigeria
Source: PLoS One. 2025 Dec 15;20(12):e0338739. doi: 10.1371/journal.pone.0338739 (PMC12704850; doi:10.1371/journal.pone.0338739)
Supplement: S2 Fig — Notes: Overview by State and Local Governmental Areas. (A) Pharmacy locations in the Federal Capital Territory (FCT). Urban pharmacies (green) are densely concentrated in the city center, with rural pharmacies (orange) more dispersed. (B) Pharmacy locations in Lagos State. Urban pharmacies (green) are clustered around the Lagos metropolitan area, while rural pharmacies (orange) extend eastward and inland. (C) Pharmacy locations in Rivers State. Urban pharmacies (green) are concentrated in the south, while rural pharmacies (orange) are distributed inland. (D) Pharmacy locations in Adamawa State. Urban pharmacies (green) are densely grouped in the central metropolitan zone, with rural pharmacies (orange) forming a surrounding arc. (E) Pharmacy locations in Kano State. Urban pharmacies (green) are clustered in the central part of the state, while rural pharmacies (orange) are more dispersed, extending southward and slightly to the north. (F) Pharmacy locations in Anambra State. The map shows distinct urban clustering (green) in the northern part of the state, with rural sites (orange) scattered throughout. Maps created by the authors using public data from Natural Earth. (PDF) [file pone.0338739.s002.pdf]

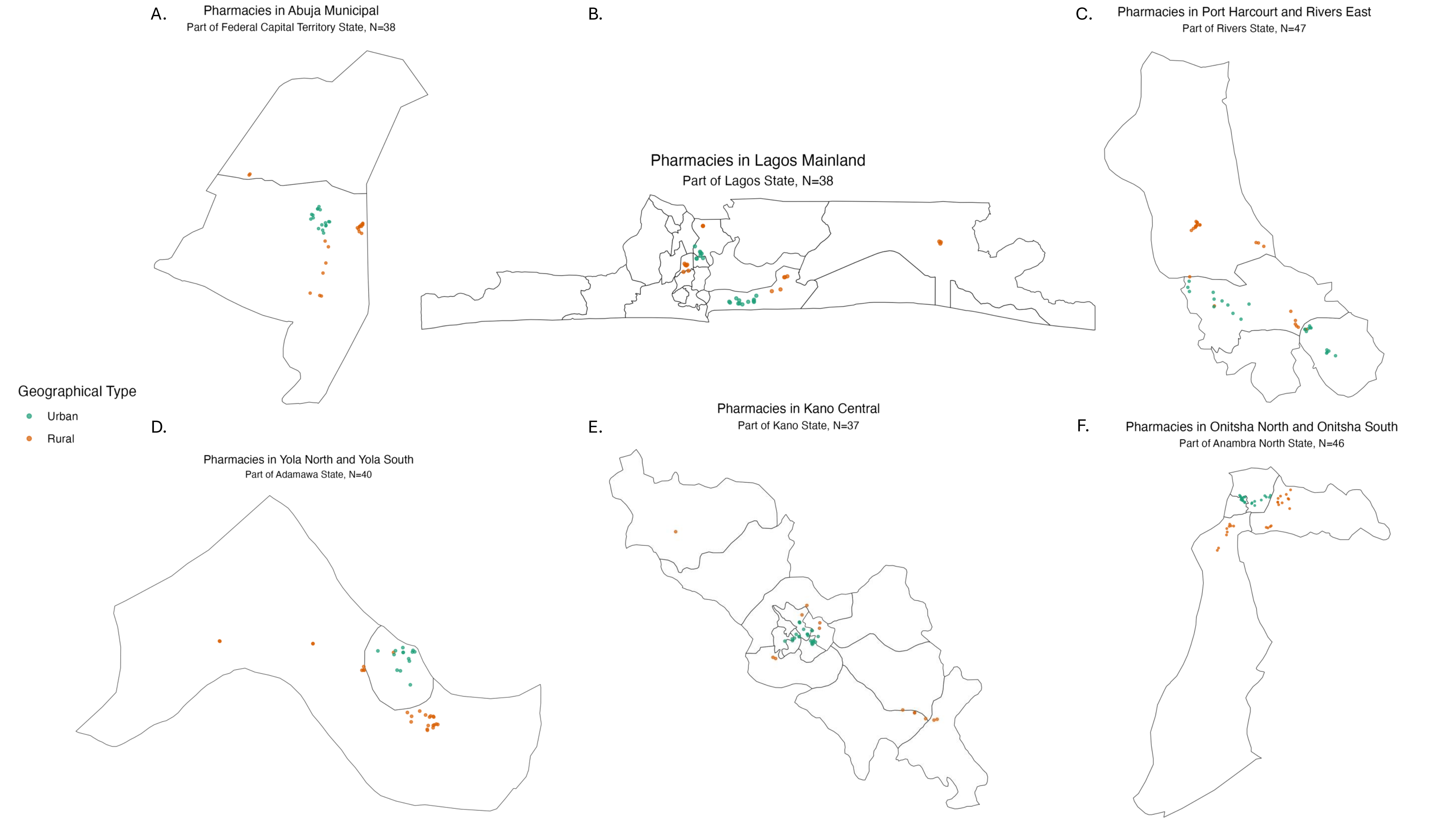
A.
Pharmacies in Abuja Municipal
Part of Federal Capital Territory State, N=38
B.
Pharmacies in Lagos Mainland
Part of Lagos State, N=38
C.
Pharmacies in Port Harcourt and Rivers East
Part of Rivers State, N=47
Geographical Type
Urban
Rural
D.
Pharmacies in Yola North and Yola South
Part of Adamawa State, N=40
E.
Pharmacies in Kano Central
Part of Kano State, N=37
F.
Pharmacies in Onitsha North and Onitsha South
Part of Anambra North State, N=46
