# Supplementary material for: Is price associated with the quality of medicines? Evidence from active pharmaceutical ingredient testing in Nigeria
Source: PLoS One. 2025 Dec 15;20(12):e0338739. doi: 10.1371/journal.pone.0338739 (PMC12704850; doi:10.1371/journal.pone.0338739)
Supplement: S1 Table — Notes: The empirical model is a probit regression, with marginal effects and standard errors exported. The outcome is defined as whether the drug non failed a laboratory test performed using High-Performance Liquid Chromatography (HPLC) due to at least a low API (below 99%). Price is measured in natural logarithm and in Nigerian Naira. All empirical models control for location (city and geographical area), drug and manufacturer type, and pharmacy size. Standard errors are clustered at the city level and reported in parentheses. *** p < 0.01, ** p < 0.05, * p < 0.1. (DOCX) [file pone.0338739.s005.docx]

| **Dependent variable: Passing the laboratory defined as non-failing because of at least one low API** | | | | |
| --- | --- | --- | --- | --- |
|  | (1) | (2) | (3) | (4) |
| Ln (price) | 0.0777 | 0.0767 | 0.0941* | 0.0936* |
|  | (0.0566) | (0.0583) | (0.0545) | (0.0556) |
| **Characteristics of pharmacies** |  |  |  |  |
| Air conditioning |  | -0.0420 |  | -0.0805 |
|  |  | (0.0520) |  | (0.0491) |
| Medicines displayed on shelves |  | 0.0155 |  | -0.0750*** |
|  |  | (0.0472) |  | (0.0268) |
| Cool-chained devices |  | 0.00597 |  | 0.0514 |
|  |  | (0.0596) |  | (0.0550) |
| Medicines exposed to direct sunlight |  | 0.111** |  | 0.144*** |
|  |  | (0.0495) |  | (0.0525) |
| Medicines placed on the floor |  | -0.0779 |  | -0.0870 |
|  |  | (0.0572) |  | (0.0739) |
| **Characteristics of drug samples** |  |  |  |  |
| Package in normal condition |  |  | 0.0747 | 0.0745 |
|  |  |  | (0.150) | (0.129) |
| Expiration date on package |  |  | 0.0104 | 0.104 |
|  |  |  | (0.0721) | (0.0802) |
| Storage temperature on package |  |  | -0.0986*** | -0.133*** |
|  |  |  | (0.0336) | (0.0451) |
| Medicines organized by brand |  |  | 0.164** | 0.192*** |
|  |  |  | (0.0674) | (0.0666) |
| **Other characteristics** |  |  |  |  |
| Kano | 0.107*** | 0.0775* | 0.0645*** | 0.0191 |
|  | (0.0234) | (0.0405) | (0.0247) | (0.0378) |
| Lagos | 0.119*** | 0.106*** | -0.0101 | -0.0731 |
|  | (0.00759) | (0.0315) | (0.0386) | (0.0624) |
| Onitsha | 0.186*** | 0.174*** | 0.124*** | 0.0772* |
|  | (0.0257) | (0.0403) | (0.00617) | (0.0416) |
| Port Harcourt | 0.187*** | 0.124 | 0.0928 | -0.00598 |
|  | (0.0621) | (0.0810) | (0.0703) | (0.0803) |
| Yola | 0.0210** | -0.00130 | -0.0261* | -0.0616*** |
|  | (0.00991) | (0.0276) | (0.0144) | (0.0216) |
| Urban | -0.0284 | -0.0293 | -0.0710* | -0.0769* |
|  | (0.0465) | (0.0556) | (0.0369) | (0.0405) |
| Medium (3-4 vendors) | -0.0313 | -0.0266 | -0.0338 | -0.0323 |
|  | (0.0607) | (0.0565) | (0.0601) | (0.0518) |
| Large (5 or more) | -0.0783 | -0.0771 | -0.0772 | -0.0927 |
|  | (0.0957) | (0.107) | (0.0889) | (0.0907) |
| Antibiotics | -0.0527 | -0.0596 | -0.0710 | -0.0853 |
|  | (0.0566) | (0.0613) | (0.0631) | (0.0690) |
| Antihypertensives | -0.380*** | -0.363*** | -0.382*** | -0.369*** |
|  | (0.0566) | (0.0540) | (0.0637) | (0.0658) |
| Antimalarials | 0.0374 | 0.0467 | 0.0321 | 0.0408 |
|  | (0.0425) | (0.0408) | (0.0331) | (0.0309) |
| Nigerian manufacturer | -0.0649* | -0.0681** | -0.0610** | -0.0650*** |
|  | (0.0357) | (0.0332) | (0.0285) | (0.0240) |
| Mean Dep. Var. | 78.45 | 78.45 | 78.45 | 78.45 |
| Observations | 246 | 246 | 246 | 246 |
| R-squared | 23.89 | 24.33 | 26.98 | 28.15 |
