# Supplementary material for: Is price associated with the quality of medicines? Evidence from active pharmaceutical ingredient testing in Nigeria
Source: PLoS One. 2025 Dec 15;20(12):e0338739. doi: 10.1371/journal.pone.0338739 (PMC12704850; doi:10.1371/journal.pone.0338739)
Supplement: S4 Table — Notes: This table presents the full survey instrument used by mystery shoppers to collect data during pharmacy visits. The tool includes modules on pharmacy identification, location (urban/rural classification, GPS coordinates), observed storage conditions, and drug sample characteristics (type, dosage, packaging, price, and manufacturer information). Enumerators completed the survey electronically using the KoboCollect App immediately after each purchase to ensure data accuracy and minimize recall bias. (DOCX) [file pone.0338739.s008.docx]

| **To fill in by enumerator** | |
| --- | --- |
| Enumerator | Name |
| Respondent number | Numeric |
| Date of the visit |  |
| Time of the visit |  |
| **Pharmacy information** | |
| Name of location/place where sample was taken |  |
|  | City [select from the list] |
| Type of location | 1. Urban |
|  | 2. Rural |
| Name of pharmacy |  |
| Street address | [Standard Form] |
| Type of pharmacy |  |
|  | 1. Retail |
|  | 2. Distributor |
| GPS coordinates | |
| Generate random list (up to 20) – select up to 3 | |
| Now that you exited the pharmacy, did you find any drugs from the previous randomized list in this pharmacy? | 1. Yes |
|  | 2. No |
| **Appearance of the pharmacy** | |
| Size of the business |  |
|  | 1. Small |
|  | 2. Medium |
|  | 3. Large |
| Do you observe any devices to store some medicine that require a cool-chain system? |  |
|  | 1. Yes |
|  | 2. No |
| If yes, please describe the devices. |  |
|  | 1. Refrigerator |
|  | 2. Cool Box |
|  | 3. Other (please specify) |
| Observe and record the following |  |
|  | 1. Drugs were display on the shelf (Yes/No/Not able to observe) |
|  | 2. Drugs bottle and boxes were display on the floor (Yes/No/Not able to observe) |
|  | 3. Drugs shelves are not able to reach by direct sunlight (Yes/No/Not able to observe) |
|  | 4. Drugs were display in the place where direct sunlight reach (Yes/No/Not able to observe) |
|  | 5. Apply air-condition system for the whole pharmacy shop (Yes/No/Not able to observe) |
|  | 6. Drugs are organized by brand/generic or type of drug (Yes/No/Not able to observe) |
|  | 7. Others (specify) |
| Are there any additional observation notes you want to record which were not included in the previous 5 observation questions (for pharmacy)? | Record here |
| **Drug information and appearance** [For each drug selected (should be 1)] | |
| **Random list of 20 drugs [select single choice for drug purchased, among the first 3** **]** | |
| Is the sample brand or generic? |  |
|  | 1. Brand |
|  | 2. Generic |
| If sample brand, name trade or brand name | [open or list] |
| Type of drug | Analgesics |
|  | Antimalarials |
|  | Antibiotics |
|  | Multivitamins |
|  | Antihypertensives |
| Sample presentation |  |
|  | 1. Quantity of tabs (number) |
|  | 2. Other (please specify) |
| Dosage |  |
|  | 1. Add dosage in mg |
| Observe and record the following |  |
|  | 1. Are the pills complete? (Yes/No) |
|  | 2. Are there broken pills? (Yes/No) |
|  | 3. Package condition (please specify) |
|  | 4. Language using on package (please specify) |
|  | 5. Price (please specify) |
|  | 6. Color pills (please specify) |
|  | 7. Odor (please specify) |
|  | 8. Other (please specify) |
| Price | [price here in Naira] |
|  |  |
| Expiration date | [Date] to select |
| Manufacturer information |  |
|  | 1. Name |
|  | 2. Address [form address] |
| Manufacturer’s Batch or Lot Number |  |
| Manufacturing date |  |
| Indicate storage temperature per manufacturer’s labeling and instructions | [temperature here] |
| NAFDAC Registration or licensed number | [registration number here] |
| Notes | [Please write any additional information you consider relevant.] |
