# Supplementary material for: Is price associated with the quality of medicines? Evidence from active pharmaceutical ingredient testing in Nigeria
Source: PLoS One. 2025 Dec 15;20(12):e0338739. doi: 10.1371/journal.pone.0338739 (PMC12704850; doi:10.1371/journal.pone.0338739)
Supplement: Attachment — Submitted filename: PLOS_Reviewers.docx [file pone.0338739.s010.docx]

**Manuscript ID:** PONE-D-25-41093

**Title:** *Is Price Associated with the Quality of Medicines? Quantitative Evidence from Nigeria*

**Response to Reviewers – Resubmission to PLOS ONE**

We thank the editors and reviewers at *PLOS Global Public Health* for their constructive feedback. We carefully revised the manuscript based on their comments, which have helped improve the clarity, robustness, and transparency of our study. Below, we provide a detailed, point-by-point response to each comment, with an indication of how we addressed them in the revised manuscript submitted to *PLOS ONE*.

**Reviewer 1:**
General comment:
The article presents a research work investigating the relationship between the price and quality of medicines in Nigeria, where SF medicines pose a significant health risk and highlighting the challenges of determining medicine quality in LMICs. It is a well-organized article and written in a clear language and with somewhat of an interesting finding. The authors have cautioned against the direct translation of their findings into practice citing the potential limitations of the study. They promoted strengthening of regulatory oversight, increasing frequency of drug quality inspections, and promoting authentication technologies to empower consumers to make more informed choices.

Specific comments:

- Page 6, Paragraph 1: It is not clear how the authors came up with the sample size of 1,214. It would be good to say something about it.

We clarified our sampling procedure in the revised manuscript. Specifically, we added the following explanation:

*“We aimed to purchase around 200 drugs per city, for a total of almost 1,200 drug samples, evenly split between rural and urban areas. This way, we stratified our sample by city and geographical area. In 93.7% of the visits, the enumerators successfully found the first drug on the list (N = 1,214).”*

- Page 7, Paragraph 1 – Could the reason why the sub-sample for analysis excluded multivitamins partly be due to the complex analysis procedure required for multivitamins? If so, it is best to mention it as one of the reasons.

The exclusion of multivitamins was primarily due to their low prevalence in our sample. To reflect this, we revised the text as follows:

*“This statistically representative sub-sample was selected using a weighted average by type of drugs of the total 1,124 purchased medicines, excluding multivitamins, which were uncommon in the pharmacies sampled and would not allow for enough variation for analysis (see [24] for more information).”*

- Page 16, Paragraph 1 – “In the Nigerian context, observable indicators such as medicines being exposed to direct sunlight (as proxy for being displayed in more public areas), being organized by brand, and having expiration dates clearly displayed on the packaging, may serve as signals of higher quality.” I am wondering how exposure to direct sunlight could be a sign of higher quality. We know light, moisture and temperature have detrimental effects on drug stability. Afterall, the recommended storage condition is ‘in a cool, dry place and away from direct sunlight.’ How is this explained? Could it be coincidental? “Similarly, pharmacies having medicines displayed on shelves and drug samples featuring storage temperature information on the packaging may act as proxies for poor quality.“ It is somewhat paradoxical why displaying medicines on shelves and stating storage temperature on packaging are indicators of poor quality. I would assume these are the standard practices. If otherwise, authors should explain as this can be confusing to readers, i.e., Shelves as opposed to what? Storage temperature on packaging as opposed to where?

We recognize that these findings are counterintuitive. Ex-ante, we also expected storage conditions, temperature shown on the package, and products displayed on the shelves to be likely proxies of high quality. However, this is not what we found empirically. We have now revised the manuscript to include a detailed discussion of these unexpected associations. Specifically, we now state in our results section:

*“In terms of pharmacy characteristics, contrary to our expectations, we find that medicines exposed to direct sunlight are more likely to pass the laboratory quality test (column 2, p < 0.01), while no other pharmacy characteristics show a significant association with quality. Although this result may seem counterintuitive, anecdotal reports suggest that higher-quality medicines may be kept more visibly in public-facing positions, such as near windows or displays exposed to sunlight, to avoid detection during audits. Further, when controlling for all observable factors (column 4), medicines displayed on shelves or those purchased from pharmacies with air conditioning are less likely to meet quality standards. One possible explanation is that visually appealing pharmacies may place greater trust in their suppliers or make stocking decisions based on market preferences and aesthetics than on rigorous quality assurance practices. This could explain why medicines displayed on shelves or in air-conditioned pharmacies show lower quality, despite appearing more professional to consumers.*

*Regarding drug sample characteristics, contrary to our expectations, we find that indicating a temperature on the package is negatively associated with quality (columns 3 and 4). Notably, 18% of samples did not specify a precise storage temperature but included a generic statement such as “do not store above 30°C.” This may suggest that certain labeling practices serve more to signal compliance than to reflect actual storage or handling. In contrast, packages that display an expiration date and those from pharmacies that organize medicines by brand are associated with a 14.0 and 10.9 percentage point higher probability, respectively, of passing the laboratory test (column 3). These associations are robust and even stronger in magnitude when controlling for all other observable characteristics (column 4). These features may reflect more rigorous manufacturing standards or inventory management practices.”*

**Reviewer 2:**

General comment:
This manuscript addresses an important and timely public health issue: whether medicine price can serve as a reliable proxy for quality in low- and middle-income countries (LMICs), using Nigeria as a case study. The topic aligns well with the scope of PLOS Global Public Health and is of high relevance to researchers, regulators, and policymakers working in pharmaceutical safety and health equity. While the study provides valuable empirical evidence, several methodological and interpretive concerns limit its current contribution.

Specific comments:

**Strengths**

- The study investigates a real-world, underexplored issue in pharmaceutical markets in LMICs.
- The use of a mystery shopper approach and data from over 1,200 pharmacies across diverse regions strengthens the external validity.
- Laboratory quality testing (HPLC) provides an objective outcome measure of drug quality.

**Weaknesses**

- Only 246 out of 1,214 purchased medicines were tested via HPLC, raising concerns about selection bias; no formal test for representativeness is presented.

We understand the concern about representativeness. We selected the laboratory-tested sub-sample using weighted averages based on the availability of drugs in the pharmacies visited by the enumerators. This ensured that the sub-sample (N=246) tested in the laboratory reflected the distribution of drugs in the full sample (N=1,124). We clarified this in the manuscript as follows and invite the reviewer to see more information in [24]:

*“This statistically representative sub-sample was selected using a weighted average by type of drugs of the total 1,124 purchased medicines, excluding multivitamins, which were uncommon in the pharmacies sampled and would not allow for enough variation for analysis (see [24] for more information).”*

- The study design is observational, yet the interpretation implies causal inference.

We revised the manuscript throughout to remove language implying causality and instead emphasize associations. For example, we now use phrases like “associated with” or “correlated with” rather than “effects of” or “impact of.” Our discussion now has a limitation section explicitly noting:

*“Our study is not without limitations. First, while we establish associations between price and quality of medicines, we cannot infer causal relationships from our results.”*

- Some results are counterintuitive and not sufficiently explained (e.g., drugs exposed to sunlight having higher quality).

Please see revised interpretation of the results.

*“In terms of pharmacy characteristics, contrary to our expectations, we find that medicines exposed to direct sunlight are more likely to pass the laboratory quality test (column 2, p < 0.01), while no other pharmacy characteristics show a significant association with quality. Although this result may seem counterintuitive, anecdotal reports suggest that higher-quality medicines may be kept more visibly in public-facing positions, such as near windows or displays exposed to sunlight, to avoid detection during audits. Further, when controlling for all observable factors (column 4), medicines displayed on shelves or those purchased from pharmacies with air conditioning are less likely to meet quality standards. One possible explanation is that visually appealing pharmacies may place greater trust in their suppliers or make stocking decisions based on market preferences and aesthetics than on rigorous quality assurance practices. This could explain why medicines displayed on shelves or in air-conditioned pharmacies show lower quality, despite appearing more professional to consumers.*

*Regarding drug sample characteristics, contrary to our expectations, we find that indicating a temperature on the package is negatively associated with quality (columns 3 and 4). Notably, 18% of samples did not specify a precise storage temperature but included a generic statement such as “do not store above 30°C.” This may suggest that certain labeling practices serve more to signal compliance than to reflect actual storage or handling. In contrast, packages that display an expiration date and those from pharmacies that organize medicines by brand are associated with a 14.0 and 10.9 percentage point higher probability, respectively, of passing the laboratory test (column 3). These associations are robust and even stronger in magnitude when controlling for all other observable characteristics (column 4). These features may reflect more rigorous manufacturing standards or inventory management practices.”*

- Potential confounders — such as brand origin, manufacturer, or source of supply — are not accounted for.

We now include manufacturer origin (Nigerian vs. international) as a covariate in all models. We found that most of the manufacturers (63.35%) of the drugs purchased were Nigerian. The drug manufacturer being Nigerian is also negatively and statistically significantly correlated (at 90% confidence level) with passing the laboratory test, which is our measure for drug quality.

- The reliance on price as a sole strong predictor is problematic in a market with potential informal pricing mechanisms and supply variability.

Thank you for raising this point, which we agree with. We explicitly described in the introduction that there are several indicators that consumers can use to infer the quality of drugs, price being one. However, our main hypothesis to test is whether price is associated with the quality of medicines, conditional on other signals consumers can use.

We now address your point on price potentially being a predictor, but not a perfect proxy, in the discussion as follows:

*“However, relying solely on price as a quality signal may be difficult, especially in markets characterized by imperfect competition and price differentiation [29]. While our results show that very low prices are often associated with poor-quality medicines, they also reveal that price is not a perfect indicator: in our sample, 33.9% of medicines in the highest price bin (>NGN 2000) still failed the quality test. Market variability, informal pricing, and branding may all complicate the relationship between price and quality for certain medicines.”*

- The manuscript lacks a transparent data sharing plan in line with PLOS policies ("data available upon request" is insufficient).

We revised our data sharing plan to comply with PLOS standards. The manuscript now includes:

*“Laboratory results for the subsample of tested medicines (N=246) are available in the Supplementary Material of the following publication: Maffioli EM and Anyakora C. “A Comparative Study Between Near-Infrared (NIR) Spectrometer and High-Performance Liquid Chromatography (HPLC) on the Sensitivity and Specificity.” PLOS ONE. 2025. DOI:* [*https://doi.org/10.1371/journal.pone.0319523*](https://doi.org/10.1371/journal.pone.0319523)*”*

- Ethical considerations regarding the use of mystery shoppers are not adequately addressed.

This study was approved by the University of Michigan (HUM00214684) and the Health Research Ethics Committee (HREC) in Nigeria (NHREC/01/01/2007). We added the following section after ethics approval:
***“Human Ethics and Consent to Participate declarations****: Not applicable. This study does not entail human subject research. Verbal or written consent does not apply.”*
