# Supplementary material for: Is price associated with the quality of medicines? Evidence from active pharmaceutical ingredient testing in Nigeria
Source: PLoS One. 2025 Dec 15;20(12):e0338739. doi: 10.1371/journal.pone.0338739 (PMC12704850; doi:10.1371/journal.pone.0338739)
Supplement: Attachment — Submitted filename: Editors comment_PONE-D-25-41093.docx [file pone.0338739.s009.docx]

I have read the paper as well, and I find it interesting and well-written. I align with some of the issues raised by one of the referees. Also, I have some further comments that can help you enhance the paper and unlock its full potential.

1. Prices of products vary with local income across states as well as across neighborhoods within these states, once you account for neigbhourhood-income-specific tastes, drugs that are relatively expensive in some locations can be instead relatively cheap for the in others without any changes in quality.
2. More robustness to show that prices are driven less by cross-city variation and more by product quality, as per your assertion. Control for states as well as neighborhood characteristics. Or hold states and location constant by exploring price and quality within pharmacies within a spatial reference radius.
3. Also, it is not clear with the design the characteristics of your silent shoppers: What are the exclusion criteria? Do you have their socio-economic attributes that can be used as a vector of covariates? For example, do they negotiate prices with pharmacies? Are they or a close family member on any medications similar to what they are shopping for? Do you randomly assign them to a location where they buy, or did you use the same location they are familiar with? More clarifications on these are needed.
4. You can also control for store-level price indexes by obtaining the price of a commonly purchased commodity across Nigeria, e.g., sugar or salt, to determine whether prices are driven more by store characteristics than by drug quality.
5. I'd also love to know how seasonal fluctuations affect demand, which can impact price. Was the survey conducted in the same month across the selected states? What are the indications of temperature and humidity that can affect disease outbreaks and subsequently drug demand and price across time and states?
