# Supplementary material for: Is price associated with the quality of medicines? Evidence from active pharmaceutical ingredient testing in Nigeria
Source: PLoS One. 2025 Dec 15;20(12):e0338739. doi: 10.1371/journal.pone.0338739 (PMC12704850; doi:10.1371/journal.pone.0338739)
Supplement: Attachment — Submitted filename: Response to Reviewers.docx [file pone.0338739.s011.docx]

**Manuscript ID:** PONE-D-25-41093

**Response to Editor and Reviewers**

**Response to the Academic Editor**

I have read the paper as well, and I find it interesting and well-written. I align with some of the issues raised by one of the referees. Also, I have some further comments that can help you enhance the paper and unlock its full potential.

We sincerely thank the Academic Editor for the thoughtful and encouraging feedback on our manuscript. We are pleased that you found the study interesting and well written. We have revised the manuscript accordingly to improve its analytical robustness, clarity, and overall presentation. The specific revisions made in response to your suggestions are detailed below.

**Comment 1**. Prices of products vary with local income across states as well as across neighborhoods within these states, once you account for neighbourhood-income-specific tastes, drugs that are relatively expensive in some locations can be instead relatively cheap for the in others without any changes in quality.

Thanks so much for this insightful comment. We agree that local income levels and preferences can influence relative prices, such that a medicine that appears expensive in one location may be relatively cheap in another — not because of differences in production costs or quality, but due to variations in income or preferences. Unfortunately, our data were collected from only one location within each of the six geopolitical zones. Therefore, the most disaggregated level at which we can account for geographic variation is the city level and the urban/rural distinction, which we control for in our analysis. While we could, in principle, construct neighborhood-level indicators within each location, we only have about 200 drugs per location, which limits the variation in the data and makes it infeasible to estimate effects controlling for more disaggregated geographical factors. This is a really important point so we added the following sentence to the Discussion:

*“Third, while our analysis incorporates city-level controls within each geopolitical zone of Nigeria to capture broader spatial variation, we acknowledge that residual neighborhood-level income heterogeneity may influence medicine prices independently of product quality. Future research employing more disaggregated geographical data could better isolate and quantify these localized effects.”*

**Comment 2**. More robustness to show that prices are driven less by cross-city variation and more by product quality, as per your assertion. Control for states as well as neighborhood characteristics. Or hold states and location constant by exploring price and quality within pharmacies within a spatial reference radius.

Thank you very much for this follow-up comment. As we explained in our response to Comment 1, while we fully agree with this point, our data do not provide sufficient variation to control for geographic heterogeneity below the city level within each of the six geopolitical zones in Nigeria. Nevertheless, as noted above, our analysis includes controls for the most disaggregated geographical level available — the city level and the urban/rural distinction. We believe these controls capture some of the variation related to income levels and preferences (for example, between rural and urban areas within each city). In addition, we cluster standard errors at the city level to account for potential within-city correlation in pricing and unobserved local factors that may influence the prices of medicines.

**Comment 3.** Also, it is not clear with the design the characteristics of your silent shoppers: What are the exclusion criteria? Do you have their socio-economic attributes that can be used as a vector of covariates? For example, do they negotiate prices with pharmacies? Are they or a close family member on any medications similar to what they are shopping for? Do you randomly assign them to a location where they buy, or did you use the same location they are familiar with? More clarifications on these are needed.

We thank the editor for this important request for clarification regarding the characteristics of and procedures followed by, our mystery shoppers. We have expanded the Methods section to describe these aspects in more detail.

To give you more information, a team of 12 enumerators were trained by our local partner, Bloom Public Health, to act asas mystery shoppers. All enumerators were data collection assistants with prior experience in similar fieldwork. To ensure balanced representation, we assigned two enumerators per area type (urban/rural) in each of the six cities.

Each enumerator visited between 7 and 13 pharmacies per day, purchasing one drug sample per pharmacy following a pre-specified randomized list. Pharmacies were selected using a random-walk sampling procedure: enumerators started from a designated point in their city, recorded its GPS coordinates, and then proceeded to visit the nearest pharmacies as ordinary customers.

To preserve the natural purchasing environment and avoid bias, enumerators did not disclose their role or negotiate prices with pharmacists. Because the enumerators served as standardized data collectors rather than study subjects, we did not collect personal or socioeconomic information about them, nor data on whether they or a close family member were taking similar medications.

In total, the team visited 1,296 pharmacies and purchased 1,142 drug samples, meeting our goal of roughly 200 purchases per city, with equal representation between urban and rural areas (1:1 ratio).

We revised the following paragraph in the Materials and Methods section to clarify more details:

“*A team of 12 enumerators from Bloom Public Health acted as mystery shoppers, purchasing a randomly selected branded drug from a list of twenty medicines at each pharmacy visited (see [24] Table 1 for the full list). Enumerators underwent thorough training and were provided with a script outlining a cover story for each drug purchase. They were instructed to either retrieve the medication from the counter or interact with the pharmacist to request it if necessary. Two enumerators were assigned to each urban and rural area in the six cities in the study. Enumerators followed a random-walk sampling approach, beginning from a chosen starting point in their city and recording GPS coordinates for verification. Each visited approximately 7–13 pharmacies per day, purchasing one medicine per pharmacy without revealing the study purpose or negotiating prices. The twenty selected drugs spanned categories including analgesics, antimalarials, antibiotics, antihypertensives, and multivitamins, representing medicines with the highest market share, as reported by one of our local partners.”*

**Comment 4.** You can also control for store-level price indexes by obtaining the price of a commonly purchased commodity across Nigeria, e.g., sugar or salt, to determine whether prices are driven more by store characteristics than by drug quality.

We appreciate the reviewer’s suggestion to control for store-level price effects. Our study design, however, involved each mystery shopper purchasing only one drug per pharmacy, which precludes constructing within-store price comparisons or commodity-based price indexes (e.g., for sugar or salt). While we cannot directly control for store-level price variation, we mitigate this concern by including fixed effects for location (city and urban/rural), as explained above.

To further minimize potential bias from store-level price differences, we randomize the drug selection — before entering each pharmacy, enumerators were presented with a randomized list of 20 target drugs via the KoboCollect app and attempted to purchase the first available drug on the list. This approach prevented systematic selection of higher- or lower-priced products across pharmacies.

**Comment 5**. I'd also love to know how seasonal fluctuations affect demand, which can impact price. Was the survey conducted in the same month across the selected states? What are the indications of temperature and humidity that can affect disease outbreaks and subsequently drug demand and price across time and states?

Thank you for this comment. To minimize potential seasonal variation in drug demand and price, all data collection occurred simultaneously across all six geopolitical zones during a three-week period in from November 10th to the 30th in 2022. We therefore believe that any seasonal effects are limited. We now added these details in our Methods section.

Regarding climatic variation, November marks the beginning of the dry season in Nigeria, when rainfall is minimal, humidity levels are low, and temperatures are relatively stable nationwide. This period follows the end of the rainy season (May–October), when malaria and other vector-borne diseases typically peak. As a result, seasonal disease incidence and related fluctuations in drug demand and pricing are generally subdued in November, minimizing the risk of confounding due to climate-driven demand effects.

### **Response to Reviewer #1**

**General Comments:**

Thank you for addressing most of the original comments. Please read through and check minor typos and grammar in few instances, e.g.
Line 98-100: Write in past tense
Line 180-182: This should be written in past tense.

Dear Dr. Khuluza,

We are greatful for your valuable suggestions on the manuscript. We have carefully revised the manuscript to check for typos and grammar mistakes, in particular fixed past tense in lines 98-100 and 180 - 182.

**Response to Reviewer #2**

General comments: The study attempts to correlate price with medicine quality which is often a complicated relationship exists in reality. It was written well with good logical flow.

**Response:**

We are grateful for the reviewer’s thoughtful review and constructive criticism. We have revised the manuscript accordingly and provide detailed responses below.

**Comment 1**. Quality is assessed in terms of HPLC assay results however medicine quality is assessed in terms of many parameters based on the dosage forms. It includes but not limited to Assay, dissolution, disintegrations, tablet hardness, friability, Limit tests, endotoxin tests etc… . Thus better to take this fact in to consideration in the title too…..Hence rather than using the broader term medicine quality in the title and other places….Please use “API content” or assay value….

We thank the reviewer for this valuable comment and fully agree that our measure of quality, based on HPLC assay results, captures only one dimension of medicine quality, specifically, the active pharmaceutical ingredient (API) content. We have therefore revised the title (*“Is Price Associated with the Quality of Medicines? Evidence from Active Pharmaceutical Ingredient Testing in Nigeria”)* to clarify that our results refer to “API content” rather than the broader concept of “medicine quality.” We also more explicitly describe that quality in our work is measured by an acceptable API content (90-110% range) as tested by HPLC. We refer to this measure of quality throughout the manuscript.

**Comment 2**. What is the accreditation status of the testing lab (Hydrochrom Analytical Services Limited, a private laboratory located in Gowon Estate, Lagos). Better to include a statement about the accreditation status and quality assurance mechanisms’ deployed in the quality testing system. Hence the laboratory test results generated are reliable.

Thank you very much for this question. Hydrochrom Analytical Services Ltd is a registered private laboratory (RC-1864978) based in Lagos, and we added this in our Materials and Methods section. In particular, Hydrochrom Analytical Services Ltd is reputable research lab that supports research especially for Universities across Lagos. Like most research lab it has not pursued ISO 17025 but it has strong quality assurance mechanism and some of the quality assurance procedures performed for this work include:

1. Validation of analytical method was performed with the following parameters: Linearity Range, Precision (area and retention time), LOD and LOQ - all within acceptable limits.

2. Duplicate extractions were performed with amount deviation values <3.0%. In a case where deviations in amounts >3.0% are observed, another extraction is performed until consecutive extractions have values of deviation <3.0%.

3. In case of changes in retention time after excessive injections of sample solutions, a primary standard solution injection is made to reconfirm retention shifts.

4. First injection of the every analytical procedure is a primary standard solution to validate retention time.

We also invite you to read more details on the testing procedures in a related publication: Maffioli, E.M. and Anyakora, C., 2025. A comparative study between Near-Infrared (NIR) spectrometer and High-Performance Liquid Chromatography (HPLC) on the sensitivity and specificity. PloS one, 20(3), p.e0319523.

*“We conducted HPLC analysis at Hydrochrom Analytical Services Limited, located in Gowon Estate, Lagos. Drug samples were initially collected at our partner’s office in Abuja, categorized, and then shipped to the laboratory. The HPLC analysis was performed on an Agilent 1100 HPLC system equipped with an online degasser, variable UV detector, quaternary pump, autoliquid sampler, and a thermostated column compartment. Chromatographic data were processed using Chemstation Rev. B.04.03-SP1 software. A validated method was employed for each molecule, depending on its specific requirements. Prior to each analysis, system suitability was confirmed using a reference standard for each analyte.* [*S1 Table*](https://journals.plos.org/plosone/article?id=10.1371/journal.pone.0319523#pone.0319523.s001) *presents the analytical parameters for linearity, correlation and detection limits of compounds by HPLC.* [*S2 Table*](https://journals.plos.org/plosone/article?id=10.1371/journal.pone.0319523#pone.0319523.s002) *describes the sample preparation and analytical conditions for each analyte.”*

**Comment 3**. Price is presented in Nigerian Currency, Naira, only in table 1 and other figures. It is better if it is converted to more international currency like the US dollar for easy grasp by international readers.

Thank you very much for this comment. We have added prices in USD in Table 1 (based on the exchange rate of Nigerian Naira 438.6 = USD 1 (Nigerian Central Bank) on November 30, 2022 which refers to the end date of data collection. We included USD conversions in the footnotes of Table 1 and in Figure 2 and S1 Figure 3 for price variables, also updating the figures so the X-axis says “Price bins (Nigerian Naira)”. Given the fluctuations in exchange rates, we believe it is important to also retain prices in Nigerian Naira. As you suggested, we have therefore added the USD values.

**Comment 4**. Better to employ appropriate statistical tests for the statement of significance about the issue mentioned in lines 243-244 “Overall, the average price of drug samples that did not fail the laboratory test (NGN 1620) slightly higher than that of those samples that failed (NGN 1530)”.

Thank you for the suggestion. We have added the *p*-value for a *t*-test comparing the price (ln) difference between samples that failed and those that did not fail in the text (two-sided p-value=0.02, 95%CI [−0.37, −0.03]).

**Comment 5**. The detailed analyses still did not demonstrate price and quality direct relationships ( increase in price ..good quality medicines) as mentioned in different places throughout the text… as seen in lines 275-281. Hence better to include such observations in the abstract and the conclusions thus readers can grasp the relations between prices and quality are not direct…….but also variable among therapeutic categories….*“The bimodal shape of the distribution is more pronounced for antihypertensives and antimalarials. In contrast, the distributions for analgesics and antibiotics are more spread out, suggesting that price may be more strongly associated with the quality of these two categories of medicines than the quality of antihypertensives and antimalarials. We also observe a similar pattern when plotting the percentage of samples by price bins, for each category of medicines (S1 Fig 3). Among analgesics, the failure rate is concentrated in the lowest price bin (“Bins”.)*

Thank you very much for this comment. We fully agree that the relationship is not direct or causal, and we have clarified this point in the discussion as the first limitation. We also reviewed the text to ensure that we consistently refer to associations rather than (direct/causal) relationships throughout the manuscript.

**Comment 6**. Nigerian NAFDAC is at Maturity Level III (ML3) by WHO…… it implies the regulatory authority is mature enough to protect public health in Nigerian market. But ML3 is not achieved in many sub-Saharan African countries. Hence some of the descriptions that are mentioned in the text like “regulatory oversight is limited, infrastructure for quality testing is often inadequate”. These statements might apply in general context of Africa but might not be in Nigeria based on regulatory Maturity level….. Thus it either better to mention the NAFDAC’s Maturity level or contextualize the bold statements mentioned throughout the manuscript based on WHO evaluation bench marking mechanism of the Nigerian NAFDAC.

Thank you for this valuable comment. We appreciate the reviewer’s observation regarding Nigeria’s regulatory maturity. Indeed, the National Agency for Food and Drug Administration and Control (NAFDAC) achieved WHO Global Benchmarking Tool (GBT) Maturity Level 3 in 2022, indicating that it has a stable, well-functioning, and integrated regulatory system capable of ensuring the quality, safety, and efficacy of medical products. We have now mentioned this achievement in the Introduction. At the same time, we note that while Maturity Level 3 reflects strong national regulatory capacity, practical challenges in enforcement, post-market surveillance, and laboratory infrastructure may still exist—particularly in decentralized or private retail settings. We have therefore revised the text in the abstract as follows:

*…Determining the quality of medicines remains a challenge, particularly in low- and middle-income countries, where regulatory where regulatory capacity and enforcement vary, and resources and infrastructure for quality testing are often constrained..*

We also acknowledge the gaps in the local implementation (rather than to NAFDAC’s overall institutional capability) in the discussion as follows:

*…Strengthening the implementation of regulatory oversight at the local level, expanding routine drug quality monitoring, and promoting authentication technologies could help improve enforcement and empower consumers to make more informed choices.*

**Comment 7**. Why medicine organization by brand is emphasized in this study? It is known that medicines in pharmacy can also be organized by therapeutic class or alphabetical etc?

Thank you for this helpful comment. We emphasize organization by brand because, during our field visits to pharmacies, we observed that medicine shelving was sometimes arranged by brand rather than by therapeutic class or alphabetically, making brand-based organization a meaningful store-level characteristic in this setting. Moreover, retail merchandising literature supports the idea that “brand blocking” — grouping products by brand on a shelf — influences consumer behavior and pricing strategies. For example, Deng et al. (2016) show that displays blocked by brand (versus by sub-category) affect how shoppers process assortments and make choices. Accordingly, we include brand-based organization as a variable because it captures a plausible pathway through which store practices may correlate with both pricing and product quality (as measured by API content via HPLC). Furthermore, we emphasize organization by brand because brands correspond to specific manufacturers, and therefore products within a brand share similar production processes and quality control standards. Distinguishing medicines by brand may thus help capture variation related to manufacturing quality, complementing our focus on storage practices.

Reference

Deng, X., Kahn, B.E., Unnava, H.R. and Lee, H., 2016. A “wide” variety: Effects of horizontal versus vertical display on assortment processing, perceived variety, and choice. Journal of Marketing Research, 53(5), pp.682-698.

**Comment 8**. Although direct sun light, cold chain and other specialized storage conditions are mentioned the chemistry and storage requirements of the selected 20 medicines is not described in the manuscript in order to grasp the relationships….

Thank you for your comment. None of the 20 medicines in our sample requires a cold chain. As described in the manuscript, 18% of the samples did not specify a precise storage temperature but included a generic statement such as “do not store above 30°C.” However, none of the drugs should be stored under direct sunlight or at high temperatures. We have added this clarification in Study Setting and Sample.

*None of these medicines required cold-chain storage, and all were stable at room temperature under standard storage conditions (below 30°C and away from direct sunlight).*

**Comment 9**. The medicines used for laboratory data as well as in the price analyses in this manuscript are 20 brand products which are produced by specific manufacturers…. Hence as such, it will limit the purchase of different products available in each pharmacy. Hence I assume products other than these 20 brands will not be purchased. Based to include such limitations in the discussion too….

Thank you very much for this comment. We had noted this limitation in the discussion section, but we have now revised the text to highlight some of the specific details you mentioned, as follows:

*Finally, our sample is limited to 20 specific branded drugs produced by specific manufacturers and available in Nigeria among four medicine categories. As a result, our findings may not be generalizable to other drug categories or geographical settings.*


**Comment 10.** Why one product only is purchased from a pharmacy

Thank you for this insightful comment. We limited purchases to one product per pharmacy to minimize detection risk and ensure the validity of the mystery-shopping protocol. Buying multiple medicines during a single visit could have raised suspicion among pharmacy staff, potentially altering their behavior or pricing practices. In addition, the study’s objective was to capture variation across pharmacies rather than within pharmacies. By randomly selecting one medicine per outlet, we ensured independent observations across a large and diverse sample of pharmacies, providing greater external validity.
